# Dose–response assessment by quantitative MRI in a phase 1 clinical study of the anti-cancer vascular disrupting agent crolibulin

**DOI:** 10.1038/s41598-020-71246-w

**Published:** 2020-09-02

**Authors:** Andres M. Arias Lorza, Harshan Ravi, Rohit C. Philip, Jean-Philippe Galons, Theodore P. Trouard, Nestor A. Parra, Daniel D. Von Hoff, William L. Read, Raoul Tibes, Ronald L. Korn, Natarajan Raghunand

**Affiliations:** 1 Department of Cancer Physiology, Moffitt Cancer Center, SRB-4, Tampa, FL 33612 USA; 2 Department of Electrical and Computer Engineering, University of Arizona, Tucson, AZ 85721 USA; 3 Department of Medical Imaging, University of Arizona, Tucson, AZ 85724 USA; 4 Department of Biomedical Engineering, University of Arizona, Tucson, AZ 85724 USA; 5 Translational Genomics Research Institute (TGen), Phoenix, AZ USA; 6 HonorHealth Clinical Research Institute, Scottsdale, AZ USA; 7 Winship Cancer Institute, Emory University School of Medicine, Atlanta, GA USA; 8 Department of Internal Medicine II, Julius Maximilians University and Medical Center, Würzburg, Germany; 9 Imaging Endpoints, LLC, Scottsdale, AZ USA; 10 Department of Oncologic Sciences, University of South Florida, Tampa, FL USA

**Keywords:** Cancer imaging, Tumour angiogenesis

## Abstract

The vascular disrupting agent crolibulin binds to the colchicine binding site and produces anti-vascular and apoptotic effects. In a multisite phase 1 clinical study of crolibulin (NCT00423410), we measured treatment-induced changes in tumor perfusion and water diffusivity (*ADC*) using dynamic contrast-enhanced MRI (DCE-MRI) and diffusion-weighted MRI (DW-MRI), and computed correlates of crolibulin pharmacokinetics. 11 subjects with advanced solid tumors were imaged by MRI at baseline and 2–3 days post-crolibulin (13–24 mg/m^2^). *ADC* maps were computed from DW-MRI. Pre-contrast *T*_*1*_ maps were computed, co-registered with the DCE-MRI series, and maps of area-under-the-gadolinium-concentration-curve-at-90 s (AUC_90s_) and the Extended Tofts Model parameters *k*^*trans*^, *v*_*e*_, and *v*_*p*_ were calculated. There was a strong correlation between higher plasma drug ${C}^{max}$ and a linear combination of (1) reduction in tumor fraction with ${AUC}_{90s}>15.8$ mM s, and, (2) increase in tumor fraction with ${v}_{e}<0.3$. A higher plasma drug AUC was correlated with a linear combination of (1) increase in tumor fraction with ${\text{ADC}} < 1.1 \times 10^{ - 3} \;{\text{mm}}^{2} /{\text{s}}$, and, (2) increase in tumor fraction with $v_{e}<0.3$. These findings are suggestive of cell swelling and decreased tumor perfusion 2–3 days post-treatment with crolibulin. The multivariable linear regression models reported here can inform crolibulin dosing in future clinical studies of crolibulin combined with cytotoxic or immune-oncology agents.

## Introduction

Tumor vasculature differs fundamentally from normal blood vessels, presenting opportunities for selective targeting that have led to two main categories of therapeutics: antiangiogenic agents designed to prevent neovascularization, and Vascular Disrupting Agents (VDAs) that target endothelial cells and pericytes of established tumor vasculature and induce vascular collapse^1,2^. Efforts in the former category have been more successful, with FDA approval being granted to bevacizumab, sunitinib, sorafenib, lenvatinib, and multiple other antiangiogenic agents. VDAs that have entered clinical testing as anti-cancer therapeutics include, combretastatin A4 phosphate^3^, ZD6126^4^, ombrabulin^5,6^, plinabulin^7^, and crolibulin^8,9^. Clinical development of VDAs has been hampered by non-availability of effective biomarkers to identify an Optimal Biological Dose (OBD) rather than the Maximum Tolerated Dose (MTD)^10,11^. The choice of companion diagnostic depends on the mode of drug action. For example, agents targeted to genetic alterations can be guided by assays of the specific molecular aberration or frequency of target presence in a given patient’s tumor^12^, while nanoparticle drug penetration into solid tumors may be predicted by imaging biomarkers such as ferumoxytol-enhanced MRI^13^.

Several functional imaging modalities have been explored as potential biomarkers of VDAs, with Dynamic Contrast Enhanced (DCE-) MRI being the modality of choice in clinical studies^3,4^, though Koh and colleagues have also investigated Diffusion-Weighted (DW)-MRI clinically^14^. Robinson and colleagues employed susceptibility contrast MRI enhanced with an ultrasmall superparamagnetic iron oxide contrast agent to demonstrate changes in fractional tumor blood volume of preclinical tumors treated with ZD6126^15^. Mason and colleagues utilized dynamic bioluminescence imaging to measure tumor perfusion and ^19^F MRI for tumor oximetry to assess response of pre-clinical tumors to an investigational VDA^16^. In mice bearing head and neck squamous cell carcinoma tumor xenografts, Seshadri and colleagues^17^ detected early changes in tumor vasculature following treatment with crolibulin using photoacoustic imaging (PAI) and blood oxygenation level–dependent (BOLD) MRI, both being imaging techniques that are sensitive to the oxygenation status of blood hemoglobin. A significant reduction in tumor hemodynamic response to carbogen challenge 24 h post-crolibulin was reported^17^. Using BLI and ultrasound imaging, this group also reported reductions in blood flow in pre-clinical prostate cancer xenografts following crolibulin treatment^18^. Shi et al.^19^ used intravoxel incoherent motion (IVIM) DW-MRI technique to quantify microvessel perfusion in mouse tumors. They reported changes in IVIM parameters related to blood pseudo-diffusion coefficient (D*) and the perfusion fraction (f) 2 h following treatment with combretastatin A4 phosphate that were correlated with tumor volume changes 8 days following treatment.

Early-phase clinical trials of new therapeutics offer the opportunity for development of imaging biomarkers of dose–response, since in the dose-escalation stage different patients receive different doses of the drug. This can also present a challenge, in that phase 1 clinical trials often recruit patients with a variety of cancers, which increases the biological heterogeneity of the data set. In the specific case of VDAs this heterogeneity is arguably less of a limitation since the target is the vasculature of solid tumors, which may be less dependent on the cancer type than a target that is associated with the tumor cells themselves^20^. Indeed, published studies of MRI in clinical trials of VDAs tend to be on cohorts of subjects with heterogeneous cancer types. Moreover, the majority of published studies of MRI in clinical trials of VDAs have been on subjects accrued during the dose-escalation phase^3,4,7,14,21–27^. And while the VDA dose was a variable across subjects in these published MRI studies, only a minority reported explicit attempts at dose–response assessment.

Explicit dose–response assessment using regression analysis have been reported by Galbraith et al.^3^, Mita et al.^7^, and Ricart et al.^26^. Using DCE-MRI, Galbraith et al.^3^ measured perfusion related parameters in 18 tumors of different types from 18 subjects. They correlated the perfusion parameters to plasma AUC of combretastatin-A4-phosphate by univariable regression analysis. In a study of 17 subjects with advanced solid tumors treated with the VDA plinabulin, Mita et al.^7^ demonstrated a post-treatment decrease in tumor perfusion and microvascular permeability (*k*^*trans*^) relative to baseline that they correlated against drug dose by a univariable regression analysis. In another DCE-MRI study of 24 subjects with 27 heterogeneous tumors treated with VDA denibulin, Ricart et al.^26^ reported changes of perfusion related parameters and performed a regression analysis of these changes to drug dose.

In other studies, tumor response to VDAs was assessed by pooling post-treatment versus baseline changes of MRI-measured parameters over all doses. For example, in a study of 21 patients with solid tumors of heterogeneous types who were treated with a range of doses of combretastatin-A4-phosphate, Gaya et al.^27^ correlated the changes in angiogenic profiles measured on histology to changes in DCE-MRI parameters. In a combination study of combretastatin with bevacizumab, Koh et al.^14^ reported changes of tumor Apparent Diffusion Coefficient (ADC) measured by DW-MRI in 12 patients with 12 different solid tumors. Meyer et al.^22^ observed decreases in tumor *k*^*trans*^ in 9 of 12 advanced gastrointestinal carcinomas treated with combretastatin-A4-phosphate and ^131^I-A5B7. In a DCE-MRI study of 11 advanced solid tumors in 9 subjects, Evelhoch et al.^4^ identified a threshold dose of the VDA ZD6126 above which there was a 36–72% post-treatment decrease in median tumor values of the initial-area-under-the-gadolinium-concentration-curve (IAUGC) relative to baseline values. In a DCE-MRI study of 16 advanced solid tumors in 16 subjects treated with varying doses of the VDA DMXAA, Galbraith et al.^21^ reported decreases in a semi-quantitative measure of contrast agent uptake in tumors following treatment relative to baseline values. In a phase 1 dose-escalation study of the VDA CYT997, Lickliter et al.^23^ measured whole tumor *k*^*trans*^ by DCE-MRI in 11 subjects with a variety of advanced solid tumors, and identified a threshold dose above which there was a change in post-treatment tumor K^trans^ relative to baseline values that was consistent with vascular disruption. In a study of 21 subjects with advanced solid tumors who were enrolled on 6 dose levels of the VDA BNC105P, Rischin et al.^25^ measured reductions in tumor K^trans^ and IAUGC in some subjects.

Crolibulin (EPC2407) is a 4*H*-chromene analog that binds to the colchicine binding site and produces anti-vascular and apoptotic effects^8,9^. In a phase I clinical study of crolibulin (NCT00423410), DW-MRI and DCE-MRI images were acquired at baseline and 2–3 days post-drug in 11 subjects. 14 abdominal, 2 thoracic, and 1 pelvic tumors were analyzed on images acquired with repeated breathhold imaging. Quantitative maps of parameters *k*^*trans*^, plasma volume fraction (*v*_*p*_), extracellular extravascular volume fraction (*v*_*e*_), Area-Under-the-Gadolinium-Concentration-Curve at 90 s (${AUC}_{90s}$), and *ADC*, were computed as these are expected to be affected by VDAs^10,28,29^. Multivariable combinations of these parameters were identified that were correlated with the crolibulin pharmacokinetic parameters: maximum plasma concentration (drug C_max_), area-under-the-plasma-concentration-curve (drug AUC), and drug dose. Here we report multivariable DW-MRI and DCE-MRI correlates of crolibulin pharmacokinetics that are suitable for non-invasive assessment of the spatially heterogeneous response of solid tumors to crolibulin, which we believe will enable the rational design of combination trials of VDAs with cytotoxic and immune-oncology agents ^6,30,31^.

## Materials and methods

### Clinical study

This was an open-label, single arm, multi-site study of patients with advanced solid tumors or lymphoma who had failed prior therapy. The clinical trial (NCT00423410) was approved by the Institutional Review Boards of each participating institution: Scottsdale Healthcare, Scottsdale, AZ 85258, USA; University of California San Diego Cancer Center, San Diego, CA 92093, USA; Tower Oncology Research, Beverly Hills, CA 90211, USA. Informed consent was obtained from all study participants, and all human subjects research was conducted in accordance with relevant institutional and national guidelines. Subjects who consented to the MRI portion of the study received 13–24 mg/m^2^ crolibulin by either 4 h or 1 h IV infusion daily × 3, repeated on a 21-day cycle. The primary study objectives were to determine the MTD of crolibulin with the 4-h infusion protocol and to determine Dose-Limiting Toxicities (DLTs) and other safety characteristics. Additional objectives were to collect pharmacokinetic data during day 1 of cycle 1 and to perform DW-MRI and DCE-MRI pre- and post-infusion on day 2 or 3 of cycle 1 to investigate early pharmacodynamic effects on tumor vasculature.

### MR imaging study

Three sites participated in the MRI portion of the study, and imaging was done on 1.5 T (Scottsdale Medical Imaging Ltd., Scottsdale, AZ; Cedars-Sinai Medical Center, Los Angeles, CA) or 3 T (University of California, San Diego, CA) scanners. Diagnostic quality T2-weighted anatomic images, DW-MRI, T1-weighted (T1w) unenhanced 3D-GRE MRI for T1 mapping, and DCE-MRI were acquired at each site per a common imaging protocol that was supplied to each site. Single-shot EPI DW-MRI images were acquired with in-plane resolution of ~ 1.5 × 1.5 mm^2^ and slice thickness of 6 mm. The *b* values used in this study were 0 or 100, 150, 300, and 450 s/mm^2^. DW-MRI was acquired with both isotropic diffusion weighting and diffusion weighting in superior/inferior direction during a held-inhalation breathhold. The pre-contrast T1 mapping protocol required 4 pre-contrast T1w 3D-GRE images to be collected with flip angles α of 15°, 23°, 30° and 60°. Imaging protocol parameters for the 3D-GRE imaging were: 12 slices reconstructed to a matrix size of 256 × 256, slice thickness = 5 mm, TR = 5.0 ms, TE = 2.1 ms (or minimum), and α = 30°. DCE-MRI data were collected as subjects repeated a “breathe-in, breathe-out, hold” pattern, with images being collected during each held-expiration period. The DCE-MRI series comprised of 24–30 3D-GRE images collected during repeated “held exhalation” breath-holds with a temporal resolution of ~ 20 s, for a total of ~ 8 min of scanning. Gadolinium contrast (0.1 mmol/kg) was power-injected at 4 mL/s and chased with 20 mL saline at 4 mL/s after ~ 2 pre-contrast images had been collected in the dynamic series.

Image data were received from each site in DICOM format, curated, and checked for image quality and adherence to study protocol. An in-house tool developed in MATLAB (MathWorks, Natick, MA) was used for ROI delineation and all subsequent image processing steps. The images were first analyzed by a radiologist with more than 25 years of experience in abdominal imaging (RLK). In consultation with the radiologist, target tumors and Regions-Of-Interest (ROIs) within normal reference tissues were manually contoured in a blinded fashion by an imaging scientist with more than 20 years’ experience (NR). In addition to whole tumor Volumes-Of-Interest (VOIs), a second set of contours were drawn 2–3 mm inside the tumor periphery to define the tumor core and tumor rim since VDAs reportedly have differential efficacy in these sub-regions^10,31^. Manual annotations were performed both on DW-MRI and DCE-MRI. Tumor annotations on DW-MRI were done on the lowest *b* value image, while on DCE-MRI tumors were annotated on the average of the whole series.

### DW-MRI analysis

**Figure 1:**
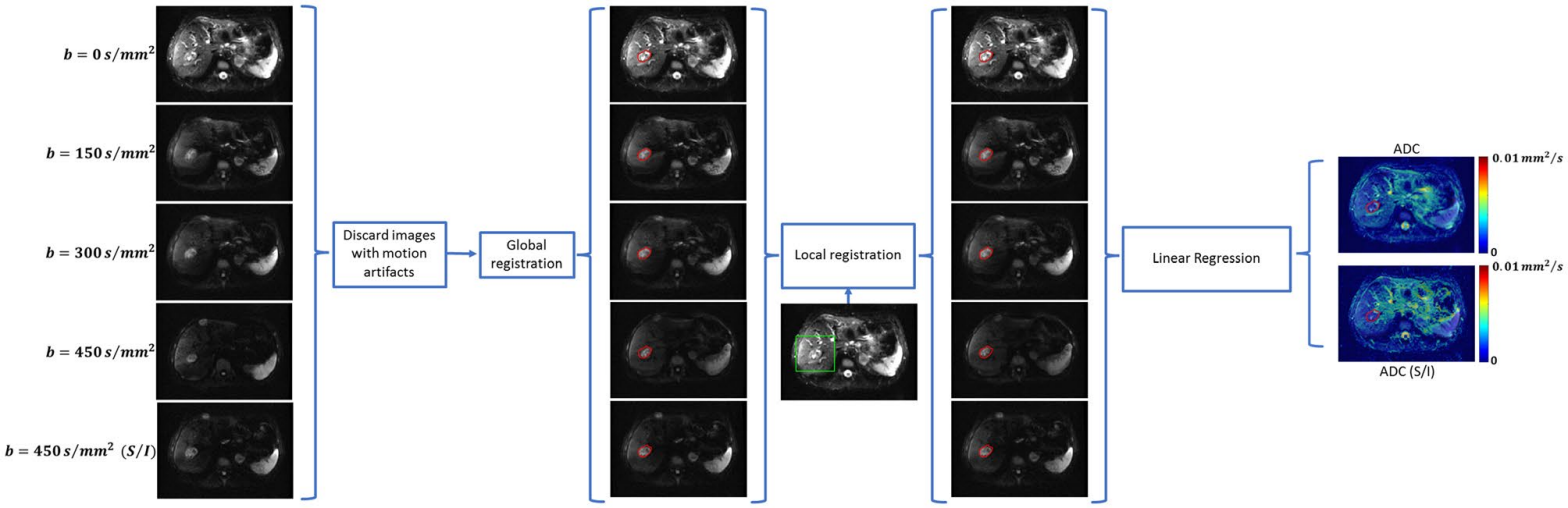
Method to extract ADC (isotropic) and ADC(S/I) from DW-MRI images acquired with multiple *b* values. A hepatic metastasis of carcinoid origin is delineated for reference. Local registration was performed in a region around the tumor (green box).

Images with significant motion artifacts were identified by visual inspection and excluded from quantitative analysis. All DW-MRI images were spatially co-registered to the lowest *b* value images. A combination of rigid and affine geometrical transformations were applied, with output quality assessed using dissimilarity metrics based on mutual information on the intensity, gradient and edge information, as described previously^32^. Following global registration, local registration was performed in a region around the manually annotated tumors ROIs. ADC maps were computed by linear regression of $\mathrm{ln}\left({\mathrm{S}}_{\mathrm{DW}}\right)=-\mathrm{ADC}\mathrm{b}+\mathrm{ln}\left({\mathrm{S}}_{{\mathrm{DW}}_{\mathrm{o}}}\right)$, where ${\mathrm{S}}_{\mathrm{DW}}$ is the image intensity and ${\mathrm{S}}_{{\mathrm{DW}}_{\mathrm{o}}}$ the non-diffusion-weighted image. An overview of the DW-MRI image processing methodology is presented in Fig. 1.

### DCE-MRI analysis

**Figure 2:**
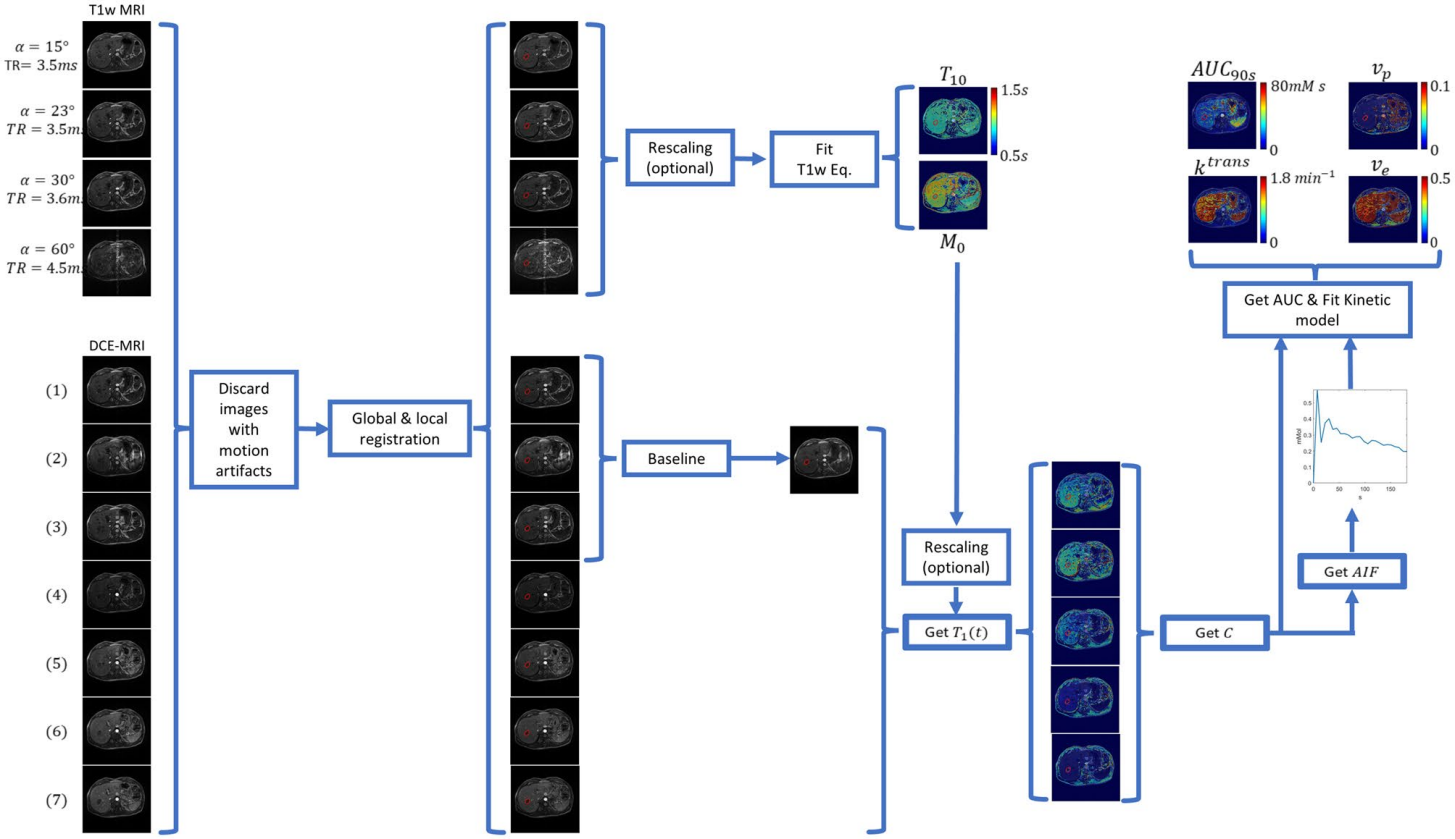
Method to extract quantitative parameters from DCE-MRI. Pre-contrast T1w images were acquired at different flip angles (α) with repetition times (TR) that were similar but not always identical. The dynamic phase typically consisted of 24–30 time points, though only 7 are depicted here for illustrative purposes. A hepatic metastasis of carcinoid origin is delineated for reference.

The pre-contrast T1w images and the DCE-MRI images were globally co-registered using the 2^nd^ post-injection time point as template due to its high contrast. This was followed by local registration in a region around the tumor and artery ROIs, the latter being defined for computation of an Arterial Input Function (AIF) required for two-compartment model pharmacokinetic analysis of the DCE-MRI^33,34^. For images collected at two of the sites, an image intensity calibration procedure was used to account for inconsistent reconstruction scaling factors between the pre-contrast T1w images (see Supplemental material 1). Pre-contrast T1 (*T*_*10*_) and proton-density (*M*_*0*_) maps were computed from the pre-contrast T1w images using the gradient-echo signal equation. In some cases additional rescaling of *M*_*0*_ was needed to account for differences in acquisition receiver gains between the pre-contrast T1w images and the DCE image series (see Supplemental material 2). The *T*_*10*_ and *M*_*0*_ maps along with the DCE-MRI images were used to calculate voxel-wise gadolinium concentrations, which were used to compute the AIF, the extended Tofts model^33^ parameters *k*^*trans*^, *v*_*p*_, and *v*_*e*_, and the model-free parameter *AUC*_*90s*_^34^ (see Supplemental material 3). An overview of the DCE-MRI image processing methodology is presented in Fig. 2.

### Tumor sub-population analysis

**Figure 3:**
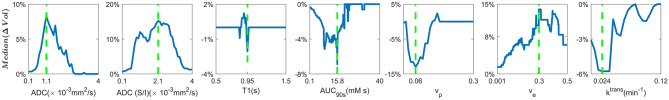
Median change in percent of tumor core volume between baseline and follow-up over all tumors in all subjects that was above (${AUC}_{90s}$, ${v}_{p}$_,_ and ${k}^{trans}$) or below (ADC, ADC (S/I), T1, and ${v}_{e}$) a rolling value for each parameter. The values that yielded the greatest absolute differences (Thres_opt_, dashed green line) were used as thresholds to calculate the “target sub-population of tumor voxels” ($\Delta Vol$) per parameter in each analyzed tumor.

To account for spatial heterogeneity in tumor response to crolibulin, we extended the work described by Chenevert et al.^35^ to each parameter computed in this study. In addition to analyzing parameter changes on a whole tumor basis, we also analyzed post-treatment versus baseline changes in tumor volume fraction ($\Delta Vol$) that was above (for ${AUC}_{90s}$, ${v}_{p}$_,_ and ${k}^{trans}$) or below (for ADC, ADC (S/I), T1, and ${v}_{e}$) a threshold value of the given parameter. For each calculated parameter we iteratively identified the threshold (Thres_opt_) that maximized the median ∆Vol over all analyzed lesions. Figure 3 illustrates per-parameter “target sub-population fraction differences” ($\Delta Vol$), with the corresponding Thres_opt_ for each parameter indicated by a green dotted line.

## Results

### Summary data

A summary of patient data and tumors analyzed is shown in Table 1. The target lesion in each subject was imaged by both DW-MRI and DCE-MRI, and a variable number of additional lesions were also visible on one or both sequences. In three subjects the DCE-MRI was not analyzable due to problems during acquisition at one of the scan dates. Two non-target tumors that were visible on DCE-MRI were not visible on DW-MRI. ADC, ADC (S/I) and T_10_ parameter maps were computed for 11 subjects, while ${k}^{trans}$, ${v}_{p}$, ${v}_{e}$, and ${AUC}_{90s}$ parameter maps were computed in eight subjects, at both scan dates. Whole tumor and tumor core VOIs were analyzed in 15 tumors on DW-MRI, with a tumor rim being clearly annotatable on 10 of them. On DCE-MRI, whole tumor VOIs were analyzed in 13 tumors, with a tumor rim being annotatable on 11 of the lesions. 11 whole tumors, eight with the tumor rim annotated, were analyzed by both DW-MRI and DCE-MRI. Quantitative analysis was performed on all the visible lesions.

**Table 1:** Summary of patient data and tumors analyzed on DW-MRI and DCE-MRI.

Subject ID	Primary cancer diagnosis	Crolibulin dose (mg/m^2^)	Length of Infusion (h)	Lesions analyzed on DW-MRI	Lesions analyzed on DCE-MRI
1	Colorectal	24	4	3	3
2	Carcinoid	24	4	1	2
3	Hemangiopericytoma	24	4	2	2
4	Hepatocellular	18	4	1	0
5	Hepatocellular	18	4	1	1
6	Colorectal	13	4	1	2
7	NSCLC	13	1	1	1
8	Ovarian	13	4	1	1
9	Leiomyosarcoma	13	4	1	1
10	Leiomyosarcoma	13	1	1	0
11	Pancreatic	13	1	2	0

### Quantitative MRI parameter maps

**Figure 4:**
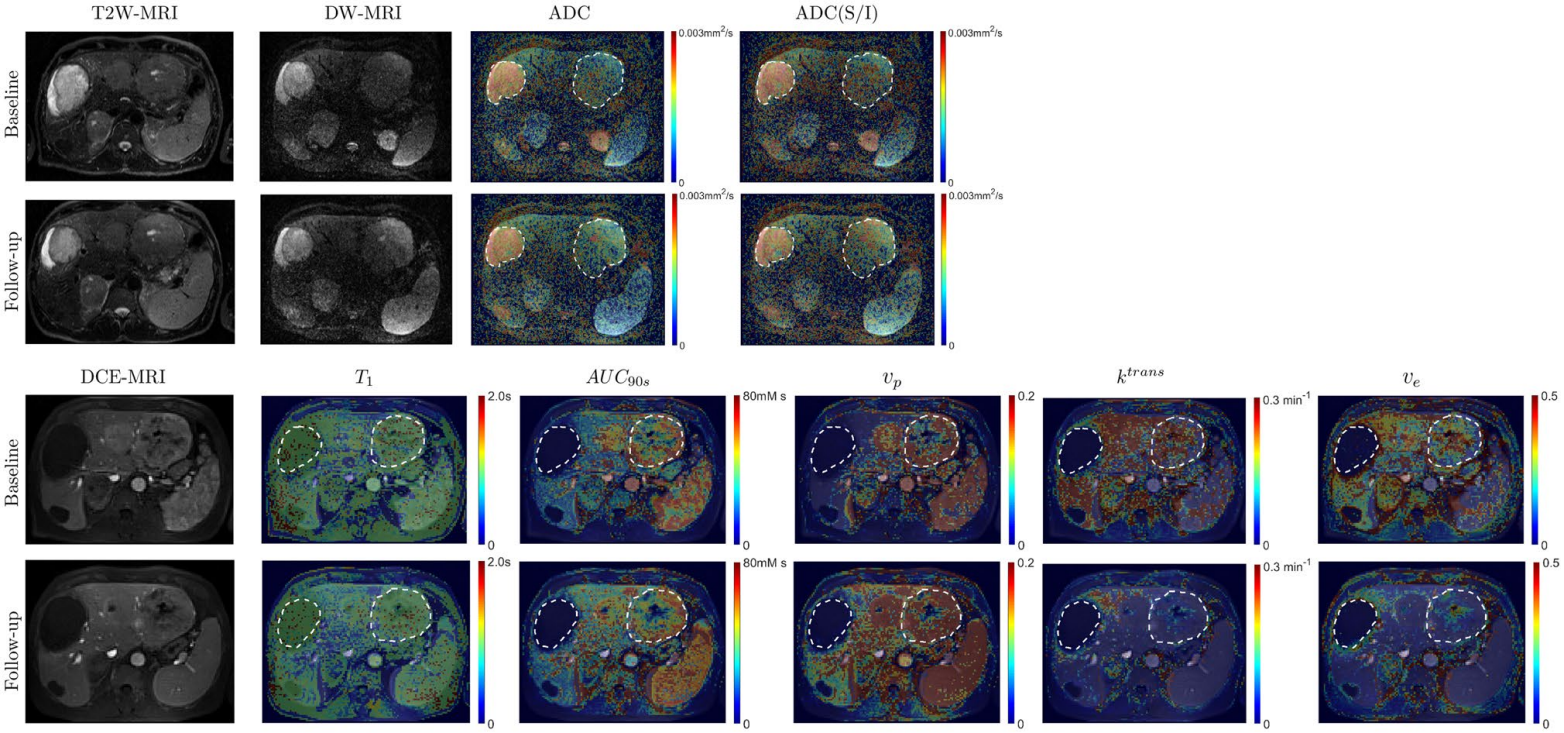
Quantitative parameters maps before and after treatment of a subject with metastatic hemangiopericytoma. There was significant replacement of healthy liver by tumor in this subject. T2-weighted fat-suppressed, DW-MRI ($b=0\,\text{s/mm}^{2}$), and DCE-MRI (2^nd^ time point post-injection of contrast) images are shown for anatomic reference, along with maps of pre-contrast T1, ADC, ADC(S/I), ${AUC}_{90s}$, ${v}_{p}$, ${k}^{trans}$ and ${v}_{e}$.

Example quantitative parameter maps before and post-treatment of a subject with metastatic hemangiopericytoma are presented in Fig. 4. Two large lesions are contoured to highlight the significant inter-lesion heterogeneity in this subject. The lesion in the right hepatic lobe is marked by hyperintensity on T2W, high ADC, and poor contrast uptake on DCE-MRI, indicating the presence of necrosis. Compared to normal paravertebral muscle and spleen, no significant changes at post-treatment relative to baseline are visible in this lesion on all the parametric maps. In comparison, the lesion in the left hepatic lobe is less intense on T2W, with a slightly shorter pre-contrast T1, and enhances on DCE-MRI. Spatially heterogeneous changes in ADC, ADC(S/I), pre-contrast T1, ${k}^{trans}$, ${v}_{p}$, ${v}_{e}$, and ${AUC}_{90s}$ are visible in this lesion at post-treatment relative to baseline (Fig. 4). Pre-treatment values of parameters computed in tumor and normal tissues are shown in Table 2.

**Table 2:** Mean ± standard error values across 11 subjects of ADC (isotropic), ADC (S/I), T1, ${AUC}_{90s}$, ${v}_{p}$, ${k}^{trans}$, and ${v}_{e}$ in various tissues at baseline.

Tissue	ADC (× 10^−3^ mm^2^/s)	ADC (S/I) (× 10^−3^ mm^2^/s)	T1 (1.5T/3T) (s)	AUC_90*s*_ (mM s)	*v*_*p*_	*k*^*trans*^ (min^−1^)	*v*_*e*_
Whole tumor	2.1 ± 0.3 (n = 15)	2.4 ± 0.2 (n = 15)	0.7 ± 0.1/0.8 ± 0.1 (n = 11)/(n = 5)	34.2 ± 7.8 (n = 13)	0.14 ± 0.04 (n = 13)	0.31 ± 0.12 (n = 13)	0.37 ± 0.10 (n = 13)
Tumor rim	1.7 ± 0.3 (n = 10)	2.1 ± 0.2 (n = 10)	0.8 ± 0.0/0.8 ± 0.1 (n = 10)/(n = 3)	39 ± 9.3 (n = 11)	0.16 ± 0.05 (n = 11)	0.35 ± 0.15 (n = 11)	0.37 ± 0.06 (n = 11)
Tumor core	1.8 ± 0.4 (n = 10)	2.1 ± 0.2 (n = 10)	0.7 ± 0.1/0.8 ± 0.1 (n = 10)/(n = 3)	36.4 ± 10.7 (n = 11)	0.15 ± 0.06 (n = 11)	0.32 ± 0.14 (n = 11)	0.17 ± 0.17 (n = 11)
Muscle	1.4 ± 0.2 (n = 11)	1.5 ± 0.1 (n = 11)	0.7 ± 0.1/0.9 ± 0.0 (n = 6)/(n = 4)	7 ± 1.7 (n = 8)	0.02 ± 0.00 (n = 8)	0.06 ± 0.02 (n = 8)	0.14 ± 0.04 (n = 8)
Spleen	1.3 ± 0.2 (n = 7)	1.5 ± 0.2 (n = 7)	0.8 ± 0.0/0.8 (n = 5)/(n = 1)	97 ± 22 (n = 5)	0.72 ± 0.16 (n = 5)	1.03 ± 1.01 (n = 5)	0.32 ± 0.29 (n = 5)
Liver	1.2 ± 0.2 (n = 8)	1.3 ± 0.2 (n = 8)	0.8 ± 0.1/0.9 ± 0.0 (n = 6)/(n = 2)	29 ± 6.6 (n = 6)	0.02 ± 0.04 (n = 6)	0.86 ± 0.42 (n = 6)	0.47 ± 0.13 (n = 6)
Renal cortex	2.9 ± 0.4 (n = 5)	2.9 ± 0.3 (n = 5)	0.9/– (n = 1)/-	95 (n = 1)	0.63 (n = 1)	0.91 (n = 1)	0.74 (n = 1)

### Reproducibility analysis

#### Tumor volume analysis

We compared tumor volumes between manually annotated VOIs on DW-MRI and DCE-MRI. We also compared tumor volumes between the two scan dates on both DW-MRI and DCE-MRI. Tumor volume correlations between scan dates were R^2^ = 0.97 (n = 15, *p* < 0.01) for tumors annotated on DW-MRI, R^2^ = 0.92 (n = 16, *p* < 0.01) for tumors annotated on DCE-MRI. Tumor volume correlations between DW-MRI and DCE-MRI were R^2^ = 0.87 (n = 14, *p* < 0.01) at baseline and R^2^ = 0.86 (n = 14, *p* < 0.01) post-treatment.

#### Reproducibility of MRI parameters in normal tissues

**Figure 5:**
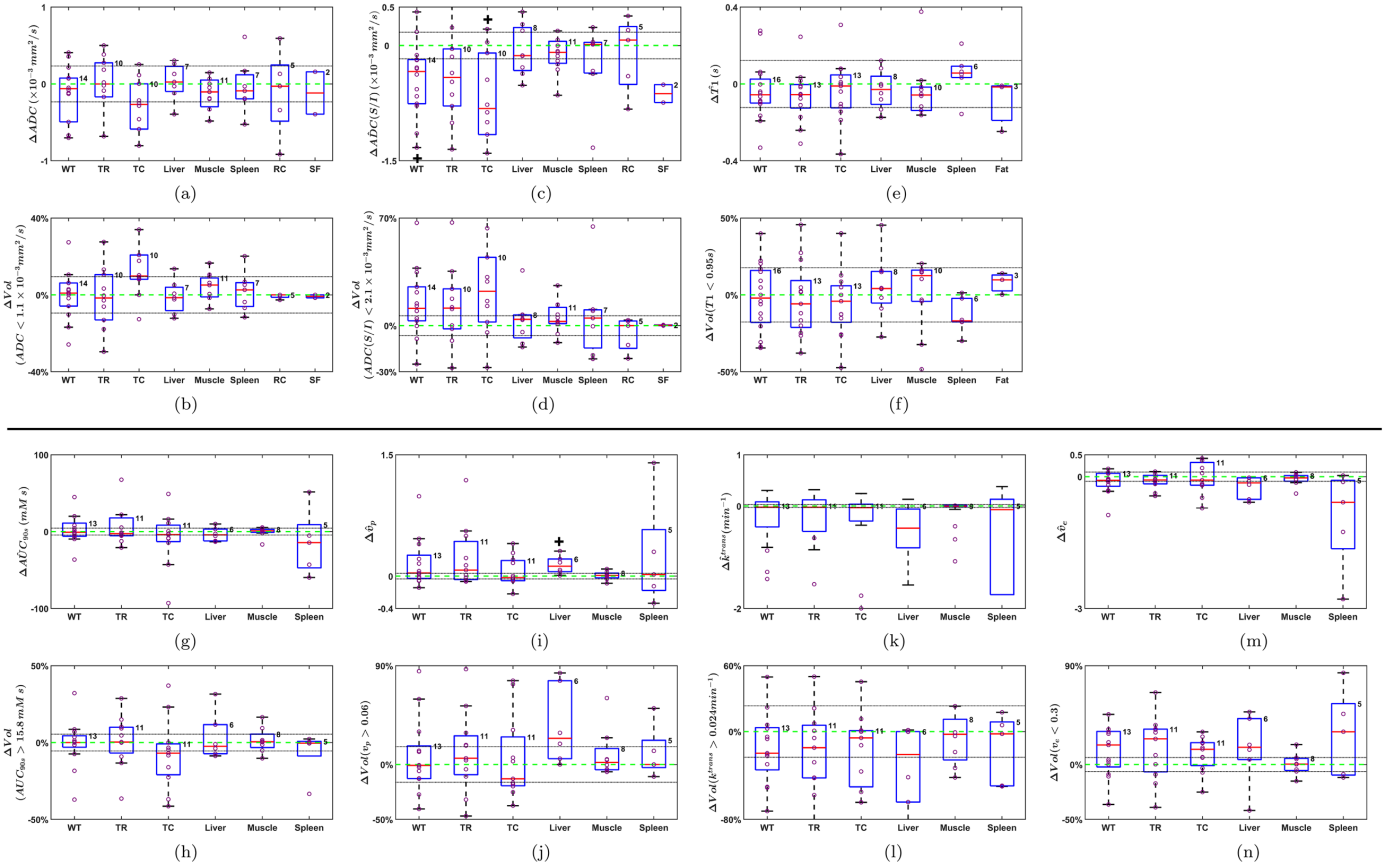
Box plots of Δ (post-treatment—baseline) changes of quantitative parameters in whole VOIs and target sub-populations of tumor voxels ($\Delta Vol$). Results for whole tumor (WT), tumor rim (TR), tumor core (TC), renal cortex (RC), and spinal fluid (SF) among others are presented. Number of samples per tissue are indicated at the right top corner of each box. Confidence intervals of muscle are depicted by dotted lines. Significant difference with respect to muscle (*p* < 0.05, Wilcoxon rank sum test) is indicated with a + above or below the box.

We compared values of the MRI parameters computed in normal tissue ROIs between baseline and post-treatment scan dates. We observed that MRI parameter changes in muscle were smaller than in other tissues, as depicted in Fig. 5. It has been reported that reproducibility of DCE-MRI parameters in muscle is similar to scan re-scan reproducibility in tumors^36^. We have summarized reproducibility in muscle tissue by the 95% Confidence Interval (CI) of the mean difference in each parameter ^14,22,27,36^, such that mean group differences outside these ranges might be related to drug effect. The CIs for the different parameters in muscle were: ± 0.2 mm^2^/s (ΔADC), ± 0.2 mm^2^/s (ΔADC(S/I)), ± 9.5% ($\Delta Vol(\mathrm{ADC})$), ± 6.4% ($\Delta Vol(\mathrm{ADC}(\mathrm{S}/\mathrm{I}))$), ± 0.1s (ΔT1), ± 17.6% ($\Delta Vol(\mathrm{T}1)$), ± 4.4 mM s (Δ), ± 5.4% ($\Delta Vol(\mathrm{ EQ AUC}\backslash \mathrm{s}\backslash \mathrm{do}5(90\mathrm{s}))$), ± 0.02 min^−1^ (Δ), ± 23% (($\Delta Vol(\mathrm{ EQ k}\backslash \mathrm{s}\backslash \mathrm{up}5(\mathrm{trans}))$), ± 0.03 ($\Delta \mathrm{ EQ v}\backslash \mathrm{s}\backslash \mathrm{do}5(\mathrm{p})$), ± 16% ($\Delta Vol(\mathrm{ EQ v}\backslash \mathrm{s}\backslash \mathrm{do}5(\mathrm{p}))$), ± 0.1 (Δ), ± 6.4% ($\Delta Vol(\mathrm{ EQ v}\backslash \mathrm{s}\backslash \mathrm{do}5(\mathrm{e}))$). These CIs are also depicted by dotted horizontal lines in Fig. 5.

### Univariable analysis of tumor response to crolibulin

Changes at post-treatment relative to baseline in the mean values of ADC, ADC (S/I), T1, ${\mathrm{AUC}}_{90\mathrm{s}}$, ${\mathrm{v}}_{\mathrm{p}}$, ${\mathrm{k}}^{\mathrm{trans}}$, and ${\mathrm{v}}_{\mathrm{e}}$, and in target sub-populations of tumor voxels (i.e., the per-parameter $\Delta \mathrm{Vol}$), are shown in Fig. 5. While muscle CIs are shown for visual reference in each panel of Fig. 5, we used the Wilcoxon rank sum test (*p* < 0.05) with respect to muscle for determining statistical significance. Relative to changes in liver, muscle and spleen, mean ADC(isotropic) tended to decrease in the tumor core following treatment (negative ΔADC values in Fig. 5a), which was also reflected in an increase in tumor volume percentage composed of voxels with ADC < 1.1 × 10^−3^ mm^2^/s (Fig. 5b), though neither trend was significant. Compared with changes in muscle there was a statistically significant decrease in mean ADC(S/I) of the whole tumor and tumor core with treatment (negative ΔADC(S/I) values in Fig. 5c), which also manifested as an increase in tumor core volume percentage comprised of voxels with ADC(S/I) < 2.1 × 10^−3^ mm^2^/s (Fig. 5d). There were no significant changes in tumor T1 with treatment, either on whole VOI basis (Fig. 5e) or $\Delta Vol$ basis (Fig. 5f). No significant pattern of change in mean tumor ${AUC}_{90s}$ was observed in whole tumor, tumor rim or tumor core VOIs (Fig. 5g), though in most tumors the volume percentage occupied by voxels with ${AUC}_{90s}$> 15.8 mM s decreased with treatment (Fig. 5h). No significant changes in ${v}_{p}$ with treatment were apparent on whole VOI basis (Fig. 5i), though in most tumor cores the percentage of voxels with ${v}_{p}>0.06$ decreased (Fig. 5j). Significant patterns of change in tumor ${k}^{trans}$ with treatment were not observable on a whole VOI basis (Fig. 5k) or $\Delta Vol$ basis (Fig. 5l). Mean changes in ${v}_{e}$ were not significant (Fig. 5m) but there was a trend towards higher volume percentages occupied by voxels with ${v}_{e}$< 0.3 in whole tumor, tumor rim and tumor core (Fig. 5n). Significant univariable correlations between $\Delta {Vol}_{{AUC}_{90s}}$, $\Delta {Vol}_{{v}_{e}}$, $\Delta {v}_{e}$, $\Delta {k}^{trans}$ and $\Delta {Vol}_{ADC}$ were observed with drug pharmacokinetic parameters (Supplemental material 4).

### Multivariable MRI correlates of crolibulin pharmacokinetics

**Figure 6:**
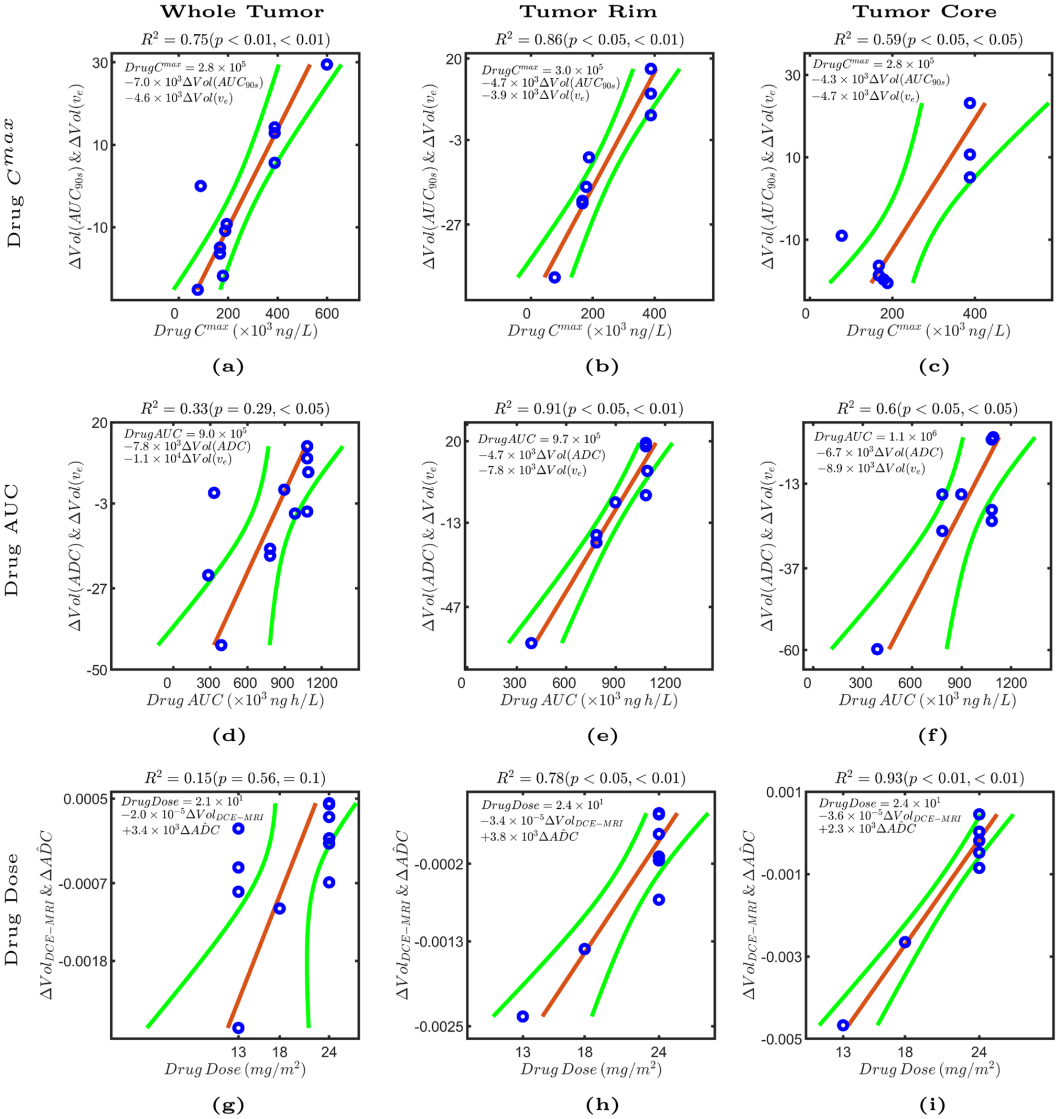
Multiple linear regression models (${\beta }_{0}+{\beta }_{1}{x}_{1}+{\beta }_{2}{x}_{2}$) shown in each panel were fitted to predict drug C^max^, drug AUC, and drug dose from pairs of quantitative MRI parameters. Each panel is an added variable plot of a drug pharmacokinetic parameter on the x-axis versus pairs of quantitative MRI parameters. Each point represents a linear combination of pairs of parameter changes on the y-axis ($\frac{{\beta }_{1}}{\Vert \beta \Vert }{x}_{1}+\frac{{\beta }_{2}}{\Vert \beta \Vert }{x}_{2}$, where ${x}_{1}$ and ${x}_{2}$ are the selected parameter changes, ${\beta }_{1}$ and ${\beta }_{2}$ their respective weight in the linear regression, and $\Vert \beta \Vert =\sqrt{{{\beta }_{1}}^{2}+{{\beta }_{2}}^{2}}$) versus crolibulin C^max^, crolibulin AUC, and crolibulin dose on the x-axis. 95% confidence bounds are shown in green and the adjusted R^2^ and *p* values for both parameters are shown above each panel. The multiple linear regression model described by the equation on the left upper corner is shown by a red line.

We investigated correlations between linear combinations of pairs of MRI parameters and crolibulin ${C}^{max}$, AUC and dose (Fig. 6). A linear combination of (1) reduction in the sub-population of tumor voxels with ${AUC}_{90s}$> 15.8 mM s, and, (2) increase in the sub-population of tumor voxels with ${v}_{e}$< 0.3, in the whole tumor was strongly correlated (R^2^ = 0.75, *p* < 0.01 both parameters) with drug ${C}^{max}$ (Fig. 6a). In eight tumors with rim and core delineated, the correlation was slightly higher for voxels in the tumor rim (Fig. 6b), and somewhat weaker for voxels in the tumor core (Fig. 6c). These observations are suggestive of higher drug ${C}^{max}$ leading to vascular collapse and possibly cell swelling with reduction of extracellular extravascular space, and consequently decreased gadolinium uptake into the tumor.

A linear combination of (1) increase in the sub-population of tumor voxels with ${\text{ADC}} < 1.1 \times 10^{ - 3} \;{\text{mm}}^{2} /{\text{s}}$, and, (2) increase in the sub-population of tumor voxels with $v_{e}$ < 0.3, on a whole tumor basis at post-treatment relative to baseline was weakly correlated with drug AUC (Fig. 6d) (R^2^ = 0.33, *p* = 0.29 ($\Delta Vol(ADC)$), *p* < 0.05 ($\Delta Vol({v}_{e})$)); the three outliers had primary diagnoses of colorectal, ovarian, and NSCLC cancer. In eight tumors with rim and core delineated, a significantly higher correlation was obtained for voxels in the tumor rim (Fig. 6e) (R^2^ = 0.91, *p* < 0.05 ($\Delta Vol(ADC)$), *p* < 0.01 ($\Delta Vol({v}_{e})$) and tumor core (Fig. 6f) (R^2^ = 0.6, *p* < 0.05 both parameters). Taken together this multivariable correlation is suggestive of increased crolibulin exposure leading to cell swelling and consequent decreases in ADC^37^ and ${v}_{e}$.

Crolibulin dose was discretized to three levels, making it challenging to discern meaningful correlations with MRI parameters (Fig. 6g). Nonetheless, we observed good correlation between drug dose and a linear combination of decreases in enhancing volume (Δ*Vol*_*DCE-MRI*_) and mean whole VOI ADC (Δ*ADC*, post-treatment − baseline) in both tumor rim (Fig. 6h) and tumor core (Fig. 6i).

## Discussion

We have demonstrated the feasibility of acquiring repeated breathhold DW-MRI and DCE-MRI in a multi-site setting in patients with advanced thoracic and abdominal tumors. We have also presented a method to extract functional parameter maps of tissue physiology from MRI image data that were acquired with slightly variable parameters on different scanners at multiple participating sites in subjects with different tumor types. The mean pre-treatment values of ADC that we computed in tumor and normal tissues are comparable to those reported in the literature for these tissues^38,39^. Quantitative parameters computed from DCE-MRI listed in Table 2 are also comparable to values reported in the literature. For example, Ahmed and Levesque^40^ reported muscle ${k}^{trans}$ of ~ 0.1 min^−1^ and ${v}_{p}$ of 0; Huang et al.^41^ reported ${v}_{e}$ of 0.1 for muscle, and ${k}^{trans}$~ 0.5 min^−1^ and ${v}_{e}$~ 0.3 in tumor; Yankeelov et al.^42^ reported ${k}^{trans}$~ 0.1 min^−1^, ${v}_{e}$~ 0.1 in muscle, and ${k}^{trans}$~ 0.25 min^−1^ and ${v}_{e}$~ 0.4 for tumor; Donaldson et al.^43^ reported ${k}^{trans}$~ 0.35, ${v}_{p}$~ 0.2, and ${v}_{e}$~ 0.2 in tumor. The generally high values of ${v}_{p}$ and ${k}^{trans}$ in the spleen in our analysis are consistent with the known physiology of this well-vascularized organ. DCE-MRI parameters computed in normal liver and renal cortex are also presented, though it should be noted that perfusion in liver parenchyma cannot truly be described using a single AIF^44^, and renal contrast agent kinetics cannot be properly described by models lacking an excretion term^45^.

MRI studies of VDAs in early-phase clinical trials have tended to assess tumor response using either DCE-MRI^3,4,7,21–27^ or DW-MRI^14^. In our study we acquired both DW-MRI and DCE-MRI on study subjects to understand the effect of crolibulin on solid tumors. Explicit dose–response assessment of VDAs by univariable regression analysis has been reported by a few groups^3,7,26^. In our study we performed multivariable regression analysis to identify pairs of MRI-measured parameters that were correlated with crolibulin pharmacokinetics. Univariable parameter changes at follow-up relative to baseline were compared against corresponding changes in muscle as a measure of reproducibility of the MRI measurements. We have also measured MRI parameter changes in several other normal tissues such as liver, spleen, kidney, and spinal fluid.

A limitation of our study is the relatively small study population, which we sought to ameliorate by analyzing multiple tumors per subject when possible. Because crolibulin pharmacokinetic parameters measured in plasma (C^max^, AUC, and dose) would be the same for all tumors in a given subject, analysis of multiple tumors per subject had the effect of making our multiple linear regression models more generalized to accommodate interlesional heterogeneity.

Another challenge was the slight heterogeneity of DW-MRI and DCE-MRI acquisition parameters across sites, which we accounted for with a robust image pre-processing pipeline (Supplementary materials 1–3). We were able to identify significant multivariable correlations despite some measurement heterogeneity in normal tissues such as muscle that would be expected to not be affected by the action of crolibulin; some of this variability is explainable by the fact that local spatial registration was only performed around tumor VOIs and arteries and not the rest of the image.

We adapted the method described by Chenevert et al.^35^ to define target sub-populations of tumor voxels as an approach to dealing with inter-lesion and intralesional heterogeneity. Crolibulin is expected to target well-vascularized tumor regions (characterized by high ${AUC}_{90s}$, ${v}_{p}$_,_ and ${k}^{trans}$) that may also support high tumor cell density (characterized by low *ADC,* ${v}_{e}$ and *T*_*10*_). In the process of identifying per-parameter Thres_opt_ as described in Fig. 3, we purposely did not utilize the per-subject crolibulin dose information in order to decrease the possibility of over-fitting in the univariable analysis (Supplementary material 6) and multivariable regression models (Fig. 6).

Gourdeau et al.^9^ previously demonstrated a dose-dependent decrease in functional tumor vasculature 4 h following crolibulin treatment of mice bearing Calu-6 human lung tumor xenografts. Rich and Seshadri^17^ employed contrast-enhanced ultrasound imaging (CEUS) and two functional imaging techniques, PAI and BOLD MRI, to investigate the antivascular effects of crolibulin in mice bearing FaDu human head and neck squamous cell carcinoma xenografts. PAI and BOLD MRI revealed substantial decreases in tumor hemoglobin oxygen saturation, as well as abolishment of tumor hemodynamic response to carbogen challenge, 24 h after treatment of the mice with crolibulin compared with pre-treatment values. Further, CEUS measurements showed significant reductions in tumor perfusion following crolibulin treatment relative to pre-treatment values. This group also reported that the corresponding hemodynamic measurements in normal skin tissue were unchanged with treatment, pointing to the tumor-specificity of the antivascular effect of crolibulin^17^. In a study of orthotopic and subcutaneously implanted Myc-CaP prostate tumors in mice, Kalmuk et al. noted a significant reduction of CD31+ vascular endothelial cell clusters and microvessel counts in tumors from crolibulin-treated mice compared to control tumors. Using MRI enhanced with an intravascular contrast agent, albumin-Gd-DTPA, they measured significant decreases in contrast enhancement 24 h after crolibulin treatment in both tumor models^18^. In a follow-up study the same group measured tumor vascular function in experimental models of glioma by MRI enhanced with the blood pool contrast agent gadofosveset trisodium. In a subcutaneous mouse model of U87 human glioma they measured a ~ 40% decrease in contrast enhancement 24 h post-therapy with crolibulin, indicative of significant drug-induced vascular shutdown. They also observed evidence of disruption of the blood–brain barrier within intracranially-implanted GL261 tumors, but not normal brain parenchyma, following crolibulin treatment^46^.

In consonance with these preclinical observations, our results indicate that an early response in tumors to crolibulin exposure is a decrease in the well-perfused fraction (voxels with ${AUC}_{90s}$> 15.8 mM s), increase in tumor fraction with restricted water diffusivity (voxels with ADC(isotropic) < 1.1 × 10^−3^ mm^2^/s or ADC(S/I) < 2.1 × 10^−3^ mm^2^/s,), and decrease in gadolinium leakage space (increase in tumor fraction with ${v}_{e}$< 0.3). A decrease in well-perfused tumor fraction would be expected from the known anti-vascular mechanism of action of crolibulin. The increase in tumor fraction comprised of voxels with low ADC and low ${v}_{e}$ may be from cell swelling consequent to vascular shutdown; the rapid timing of the post-treatment imaging argues against fibrosis or cell proliferation as alternate explanations. Tumor volume changes between baseline and 2–3 days post-crolibulin were not significant on either DW-MRI or DCE-MRI, which is consistent with the expectation that VDAs do not produce frank changes in tumor volumes at early times following initiation of treatment^47^.

In future clinical studies of crolibulin combined with a cytotoxic drug or immune-oncology agent, the multivariable linear regression models we have reported here may be useful for estimating the expected single-drug activity of crolibulin on a given patient’s tumor DW-MRI and DCE-MRI parameters, given the plasma pharmacokinetics of crolibulin measured in that patient. This information may enable personalized dosing and timing of the other drug to achieve anti-tumor additivity or synergy of the combination of drugs.

## Supplementary information

Supplementary Information.

## Data Availability

De-identified MRI images collected in this clinical trial can be made available to interested investigators via an appropriate inter-institution Material Transfer Agreement.
