# Supplementary material for: Dose–response assessment by quantitative MRI in a phase 1 clinical study of the anti-cancer vascular disrupting agent crolibulin
Source: Sci Rep. 2020 Sep 2;10:14449. doi: 10.1038/s41598-020-71246-w (PMC7468301; doi:10.1038/s41598-020-71246-w)
Supplement: Supplementary file 1 — Supplementary Information. [file 41598_2020_71246_MOESM1_ESM.pdf]

Dose-Response Assessment by Quantitative MRI in a Phase 1 Clinical Study of the Anti-Cancer Vascular Disrupting Agent Crolibulin

SUPPLEMENTARY MATERIALS 1-4

Andres M. Arias Lorza, PhD,[1] Harshan Ravi, PhD,[1] Rohit C. Philip, PhD,[2] Jean-Philippe Galons, PhD,[3] Theodore P. Trouard, PhD,[4] Nestor A. Parra, PhD,[1] Daniel D. Von Hoff, MD,[5] William L. Read, MD,[6] Raoul Tibes, MD, PhD,[7] Ronald L. Korn, MD, PhD,[8] Natarajan Raghunand, PhD,[1,9,]*

[1]Dept. of Cancer Physiology, Moffitt Cancer Center, Tampa, FL 33612, USA;
[2]Dept. of Electrical and Computer Engineering, University of Arizona, Tucson, AZ 85721, USA;
[3]Dept. of Medical Imaging, University of Arizona, Tucson, AZ 85724, USA;
[4]Dept. of Biomedical Engineering, University of Arizona, Tucson, AZ 85724, USA;
[5]Translational Genomics Research Institute (TGen), Scottsdale, AZ, USA; HonorHealth Clinical Research Institute, Phoenix, AZ, USA;
[6]Winship Cancer Institute, Emory University School of Medicine, Atlanta, GA, USA;
[7]Department of Internal Medicine II, Julius Maximilians University and Medical Center, Würzburg, Germany;
[8]Imaging Endpoints, LLC, Scottsdale, AZ, USA;
[9]Department of Oncologic Sciences, University of South Florida, Tampa, FL, USA.

*To whom correspondence should be addressed: Department of Cancer Physiology, Moffitt Cancer Center, SRB-4, Tampa, FL 33612, USA; Email: Natarajan.Raghunand@moffitt.org

Supplemental Material 1

T1 Parameter Fitting

The pre-contrast T_{10} was obtained by fitting the variable flip angle T1w voxel intensities to the GRE signal equation[1]:

$$S = S_0 \left(\frac{1-e^{\frac{-TR}{T_1}}}{1-e^{\frac{-TR}{T_1}} \cos\alpha} \right) \sin\alpha, \qquad \text{(A1)}$$

where TR and α are the repetition time and flip angle, respectively; S_0 is a function of the M_0, TE, and T2 We have neglected the contribution of the T2 term ($e^{-\frac{TE}{T2}} \to 1$) so for these short echo time images: $S_0 = M_0\, e^{-\frac{TE}{T2}} \approx M_0$. From the pre-contrast T1w images acquired at different TR and α, we fit the T1w GRE signal equation to get the unknown parameters T1 without contrast ($T10$), and M_0. M_0 is assumed to be invariant to the contrast agent[2], however in practice pre and post-contrast images may have been acquired using different scanner gains (against study protocol imaging instructions).

Voxel-wise surface fitting constraining $M_0(\boldsymbol{x}) \in \mathrm{R}^+$ and $T10(\boldsymbol{x}) \in [0\ 3]s$ in every voxel $\boldsymbol{x} \subset Z^3$ results in $T10$ and M_0 maps. M_0 is supposed to be a positive number and $T10$ selected range is based on most T1 values for different tissues at 1.5T and 3T[3-5]. Trust-region algorithm[6] is used for fitting, where the starting point is $T10(\boldsymbol{x}) = 1s$ which is close to the average T1 in most tissues[3-5], and $M_0(\boldsymbol{x})$ the average obtained M_0 for each T1w image assuming $T10(\boldsymbol{x}) = 1s$. Generally, in case TR is the same for all images, the variable flip angle method[7,8] which is a linearized model of Eq. A1 is commonly used for fitting; however in most visits variable TR and α were used for T1w acquisition, additionally trust-region non-linear fitting allows to constraint the solution to be inside a region which is useful to avoid unfeasible solutions.

For the pre-contrast parameter fitting, M_0 is assumed to be the same for each T1w image, however in practice each image may have a different scanner gain. In some cases, this information can be extracted from the image metadata[9]. However, in case this information is not clearly defined in the image metadata, these gain factors should be also estimated. The gain factors are estimated such that model fitting on re-scaled mean intensities in healthy tissue ROIs results in closer T1 values to literature values (see **Table A1**). Normal and pathologic tissue ROIs were manually annotated.

Table A1: Gold standard T1 values in healthy tissues obtained as the most common T1 literature values[3-5].

**Tissue Type**	**Field strength**	
	**1.5T**	**3T**
Muscle	1s	1.4s
Liver	0.57s	0.8s
Kidney	0.9s	1.1s
Fat	0.34s	0.35s
Bone	0.54s	0.58s
Spleen	1s	1.3s
Blood	1.4s	1.9s

To get the gain factors we used two approaches. In one approach, we make the assumption that the signal given by Eq. A1 using a T1 value from literature from a specific tissue is almost equal as the intensity in that tissue. This means the difference between intensities and resulting signal should be minimal. Therefore, to find the gain factors, we minimize a cost function based on the Sum of Squared Errors (SSE) between mean intensities and signal equation as a function of the gain factor plus a regularization to avoid unfeasible solutions. Having *N* pre-contrast registered T1w images $\{I_i \subset R^3 | i \in 1, \dots, N\}$, we define the mean intensity in image I_i in the manually annotated tissue *j* as $\hat{I}_{i_j}$. If per image, we assume the average M_0 in tissue *j* ($\widehat{M}_{0_j}$) is multiplied by a gain factor k_i, the cost function *f* is defined as:

$$f\left(k_1, \dots, k_N, \widehat{M}_{0_j}\right) = \sum_{i \in 1,\dots,N} \left(\hat{I}_{i_j} - S\left(k_i \widehat{M}_{0_j}, T_{1_j}, \alpha_i, TR_i\right)\right)^2 + \sum_{i \in 1,\dots,N} g(k_i), \qquad \text{(A2)}$$

where T_{1_j} is the T1 time in tissue *j* as defined in **Table A1**, α_i and TR_i the α and TR for image I_i, and g is the regularization function defined by the step type expression: $g(k) = K(tanh(k - k_{max}) + 1)$, which is a differentiable function with $K \rightarrow \infty$ and k_{max} the maximum allowed value for a gain factor. Finally, *S* in Eq. A1 is redefined using the gain factor by:

$$S\left(k_i\,\widehat{M}_{0_j}, T_{1_j}, \alpha_i, TR_i\right) = k_i\widehat{M}_{0_j}\left(\frac{1-e^{-\frac{TR_i}{T_{1_j}}}}{1-e^{-\frac{TR_i}{T_{1_j}}}\cos\alpha_i}\right)\sin\alpha_i. \qquad \text{(A3)}$$

Then, the gain factors k_i are obtained by solving $arg\,min_{k_1,\ldots,k_N,\widehat{M}_{0_j}}\, f$ using a simplex search algorithm[10].

In another method to get the gain factors, we use the principle that at higher α the T1-weighting increases. Then, we use the average image intensity in tissue *j* with the highest α (assuming $\alpha_N = max(\{\alpha_1, \ldots, \alpha_N\})$) to get $\widehat{M}_{0_j}$ assuming $k_N = 1$ and the signal equal to the intensity as $S\left(\widehat{M}_{0_j}, T_{1_j}, \alpha_N, TR_N\right) = \hat{I}_{N_j}$. Then, the rest of gain factors $\{k_i | i \in 1, \ldots, N-1\}$ are further obtained using the calculated $\widehat{M}_{0_j}$ and assuming $S\left(k_i\,\widehat{M}_{0_j}, T_{1_j}, \alpha_i, TR_i\right) = \hat{I}_{i_j}$.

Finally, images $\{I_i | i \in 1, \ldots, N\}$ are re-scaled to obtain re-scaled images (I_i^s) using the obtained gain factors by any of the two methods on any reference tissue *j* by $I_i^s = I_i/k_i$, then *T10* and *M₀* maps are obtained fitting the T1w GRE signal equation to the scaled images $\{I_i^s | i \in 1, \ldots, N\}$. If no reference tissue *j* is enforced, then the selected tissue *j* is the one that results in a minimal error between literature T1s and the obtained *T10* in all annotated tissues.

To get the scaling factor to estimate *T10* and M_0 we rescaled the T1w images that results in the lowest average error to the literature T1 values shown in **Table A1**, or no rescaling of the images so scaling factors equal one. In three patients no rescaling was applied because it was known that the same scanner factor was used in all images T1w images. In eight patients rescaling was applied in both visits. Using the mean estimated T1 value in each tissue *j* ($T_{1_j}^e$) and the literature T1 value in tissue *j* (T_{1_j}), the average T1 error ($100 \times \text{mean}_j\left(\left(T_{1_j}^e - T_{1_j}\right)/T_{1_j}\right)$) in the 8 patients (16 visits) that rescaling was necessary changed from 76.7%±13.3% without scaling to 36.5%±6.8% (p<0.01, Friedman's test) after rescaling.

References

1 Buxton, R. B., Edelman, R. R., Rosen, B. R., Wismer, G. L. & Brady, T. J. Contrast in rapid MR imaging: T1- and T2-weighted imaging. *Journal of computer assisted tomography* **11**, 7-16, doi:10.1097/00004728-198701000-00003 (1987).

2 Khalifa, F. *et al.* Models and methods for analyzing DCE-MRI: a review. *Medical physics* **41**, 124301, doi:10.1118/1.4898202 (2014).

3 Stanisz, G. J. *et al.* T1, T2 relaxation and magnetization transfer in tissue at 3T. *Magnetic resonance in medicine* **54**, 507-512, doi:10.1002/mrm.20605 (2005).

4 Bojorquez, J. Z. *et al.* What are normal relaxation times of tissues at 3 T? *Magnetic resonance imaging* **35**, 69-80, doi:10.1016/j.mri.2016.08.021 (2017).

5 Zhang, X. *et al.* In vivo blood T(1) measurements at 1.5 T, 3 T, and 7 T. *Magnetic resonance in medicine* **70**, 1082-1086, doi:10.1002/mrm.24550 (2013).

6 Conn, A. R., Gould, N. I. M. & Toint, P. L. *Trust-region methods.* (Society for Industrial and Applied Mathematics, 2000).

7 Sandmann, C., Hodneland, E. & Modersitzki, J. A practical guideline for T1 reconstruction from various flip angles in MRI. *Journal of Algorithms & Computational Technology* **10**, 213-223, doi:10.1177/1748301816656288 (2016).

8 Chang, L. C., Koay, C. G., Basser, P. J. & Pierpaoli, C. Linear least-squares method for unbiased estimation of T1 from SPGR signals. *Magnetic resonance in medicine* **60**, 496-501, doi:10.1002/mrm.21669 (2008).

9 Chenevert, T. L. *et al.* Errors in Quantitative Image Analysis due to Platform-Dependent Image Scaling. *Translational oncology* **7**, 65-71, doi:10.1593/tlo.13811 (2014).

10 Lagarias, J. C., Reeds, J. A., Wright, M. H. & Wright, P. E. Convergence Properties of the Nelder--Mead Simplex Method in Low Dimensions. *SIAM J. on Optimization* **9**, 112-147, doi:10.1137/s1052623496303470 (1998).

Supplemental Material 2

Concentration Curves

The gadolinium concentration curves *C* were obtained by:

$$C(t) = \frac{1}{r}\left(\frac{1}{T_1(t)} - \frac{1}{T_1(0)}\right), \qquad \text{(A4)}$$

where T1 times are calculated using Eq. A1, assuming S equal to the DCE-MRI image intensities: $\{I_{ti}^{DCE} \in R^3 \,|\, ti \in t_{min}, .., 0, \ldots t_{max}\}$, where $I_{t_{min}}^{DCE}$ and $I_{t_{max}}^{DCE}$ are the first and last DCE-MRI images, and I_0^{DCE} the image at time of injection. Then, we can redefine Eq. A1 as

$$I_{ti}^{DCE} = S_0^{DCE}\left(\frac{1-e^{-\frac{TR^{DCE}}{T_1(ti)}}}{1-e^{-\frac{TR^{DCE}}{T_1(ti)}}\cos\alpha^{DCE}}\right)\sin\alpha^{DCE}, \qquad \text{(A5)}$$

Where TR^{DCE} and α^{DCE} are the fixed TR and α for the full sequence, $T_1(ti)$ the T1 at time ti, and $S_0^{DCE} = k^{DCE}\, M_0$ where we assume a constant gain factor k^{DCE}. As the full DCE-MRI sequence is done in the same acquisition it is natural to assume a constant scanner gain for all images in a series. Concentration are defined after injection that is: $C\!: \{0, \ldots t_{max}\} \rightarrow \Re$. To get $T_1(0)$, instead of using I_0^{DCE} we use the average image before injection ($\hat{I}_0^{DCE}$) to get a higher SNR by: $\hat{I}_0^{DCE} = \mathrm{mean}\left(\{\mathrm{I}_{\mathrm{t_{min}}}^{\mathrm{DCE}}, \ldots, \mathrm{I}_0^{\mathrm{DCE}}\}\right)$. To get k^{DCE} we use different approaches. Approach 1: k^{DCE} is equal to the gain factor k_i from the pre-contrast T1w image I_i with the same flip angle (α^{DCE}) so $\alpha_i = \alpha^{DCE}$. In case of several α_i equal to α^{DCE} then k^{DCE} is equal to the average of all k_i with $\alpha_i = \alpha^{DCE}$. The rationale behind this is that it is likely that the optimized gain factor for acquiring DCE-MRI is the same as the pre-contrast T1w image with the same flip angle. Approach 2: the rationale of this approach is that to get comparable concentrations between visits the mean M_0 values between visits should be the same; to get this k^{DCE} equal the ratio between M_0 means.

In the three patients where no rescaling was applied to the T1w images, M_0 was not rescaled to get T1 at time *t.* In seven patients M_0 is rescaled such that we used the same scaling factor to scale the T1w image with α=30°, which is the same *α* used to acquire the DCE images. In one patient the concentration curves where still very different so M_0 was rescaled using approach 2 such that concentration curves in reference normal tissues became similar. The relaxivity to get

concentrations was obtained depending of the employed contrast agent: Magnevist, Multihance, Optimark; and field strength[1].

References

1 Shen, Y. *et al.* T1 relaxivities of gadolinium-based magnetic resonance contrast agents in human whole blood at 1.5, 3, and 7 T. *Investigative radiology* **50**, 330-338, doi:10.1097/rli.0000000000000132 (2015).

Supplemental Material 3

Kinetic model Parameters

From the concentration curves, we obtain the DCE-MRI parameter maps: $\mathrm{AUC}_{90\mathrm{s}}$, $\mathrm{k}^{\mathrm{trans}}$, v_{p}, and v_e. To get AUC_{90s}, concentration curves are interpolated to a higher resolution (Δ*t*=0.5*s*) and integrated from time of injection (*t*=0*s*) to *t*=90*s* to get AUC_{90s}. To get the other parameters we use the extended Tofts model. This model assumes two compartments in the tissue (v_p and v_e) that exchange contrast as:

$$\frac{\partial \mathrm{C(t)}}{\partial \mathrm{t}} = \mathrm{k}^{\mathrm{trans}}\left(1 + \frac{\mathrm{v_p}}{\mathrm{v_e}}\right)\mathrm{C_p(t)} - \left(\frac{\mathrm{k}^{\mathrm{trans}}}{\mathrm{v_e}}\right)\mathrm{C(t)} + \mathrm{v_p}\frac{\partial \mathrm{C_p(t)}}{\partial \mathrm{t}}, \tag{A6}$$

where *C* are the tissue concentrations defined by Eq. A4, and C_p the plasma concentrations. Then the parameters of the model are estimated by solving a linear system of equation as in Murase[1]. To get the plasma concentration C_p, this is defined by $\mathrm{C_p} = \mathrm{C_A}/(1 - \mathrm{H_{LV}})$ [2], where C_A is the artery contrast concentration (Arterial Input Function (AIF)), and $\mathrm{H_{LV}} \approx 0.45$ the large vessel hematocrit fractional[3]. The AIF is defined as the contrast concentration in a feeding artery[4]. There are two main approaches in the literature to get the AIF: population based[3,5] where AIF is represented by an invariant functional form, and measuring *C* at artery locations in the image[4]. In this work we measured *C* at artery locations using the manually annotated ROIs, then we discarded incorrect concentration curves[6], and finally AIF is given by the median curve from the remaining concentration curves.

References

1 Murase, K. Efficient method for calculating kinetic parameters using T1-weighted dynamic contrast-enhanced magnetic resonance imaging. *Magnetic resonance in medicine* **51**, 858-862, doi:10.1002/mrm.20022 (2004).

2 Koh, T. S., Bisdas, S., Koh, D. M. & Thng, C. H. Fundamentals of tracer kinetics for dynamic contrast-enhanced MRI. *Journal of magnetic resonance imaging : JMRI* **34**, 1262-1276, doi:10.1002/jmri.22795 (2011).

3 Georgiou, L., Wilson, D. J., Sharma, N., Perren, T. J. & Buckley, D. L. A functional form for a representative individual arterial input function measured from a population using high temporal resolution DCE MRI. *Magnetic resonance in medicine* **81**, 1955-1963, doi:10.1002/mrm.27524 (2019).

4 Calamante, F. Arterial input function in perfusion MRI: a comprehensive review. *Progress in nuclear magnetic resonance spectroscopy* **74**, 1-32, doi:10.1016/j.pnmrs.2013.04.002 (2013).

5 Parker, G. J. *et al.* Experimentally-derived functional form for a population-averaged high-temporal-resolution arterial input function for dynamic contrast-enhanced MRI. *Magnetic resonance in medicine* **56**, 993-1000, doi:10.1002/mrm.21066 (2006).

6 Li, X. *et al.* Sampling arterial input function (AIF) from peripheral arteries: Comparison of a temporospatial-feature based method against conventional manual method. *Magnetic resonance imaging* **57**, 118-123, doi:10.1016/j.mri.2018.11.017 (2019).

Supplemental Material 4

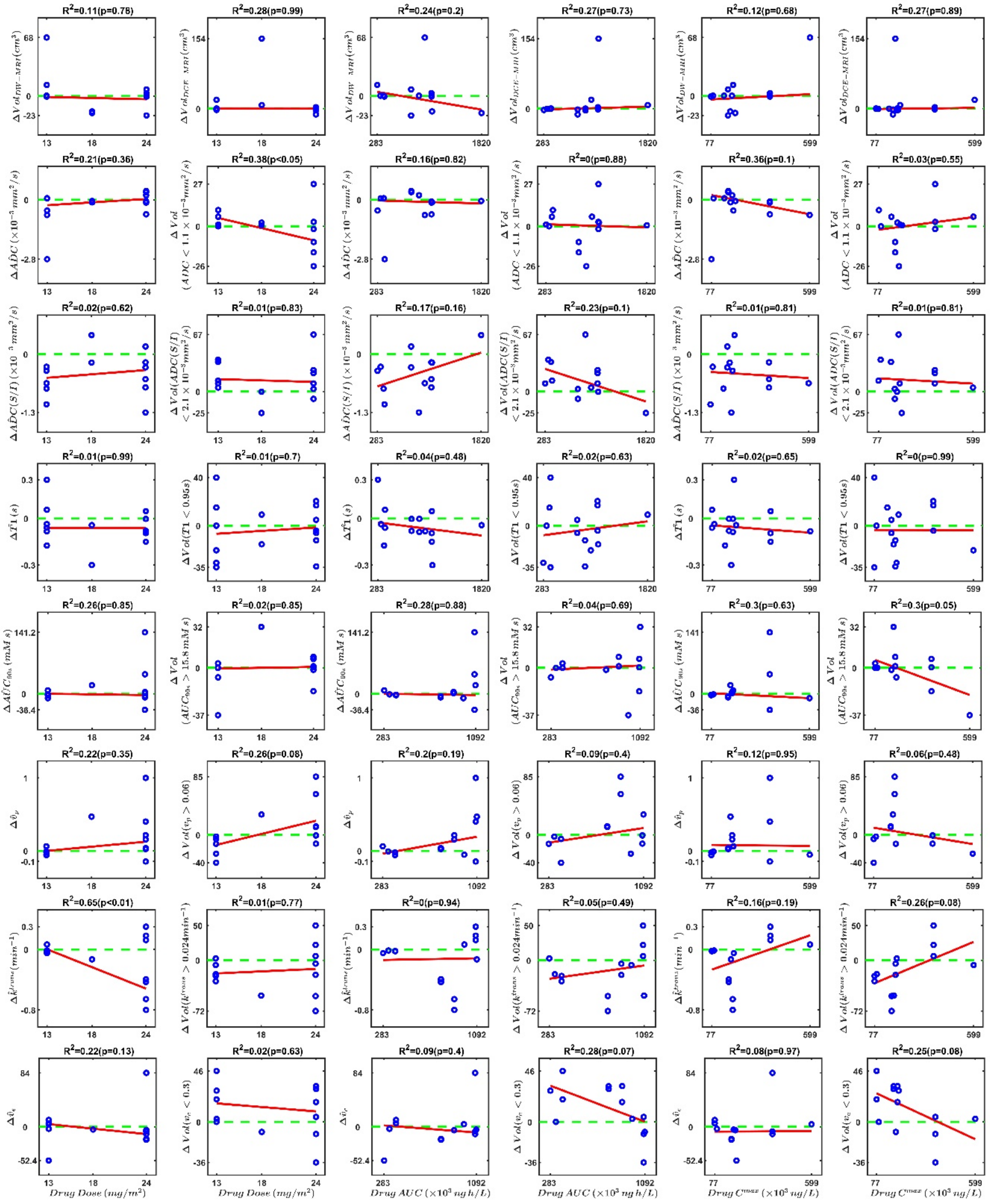

Figure A1: Linear regressions of mean changes and volume subpopulation changes at the whole tumor per parameter vs. drug dose, drug AUC, and drug C^{max}. The first row shows whole tumor volume changes annotated in DW (ΔVol_{DW_MRI}) and DCE (ΔVol_{DCE_MRI}).

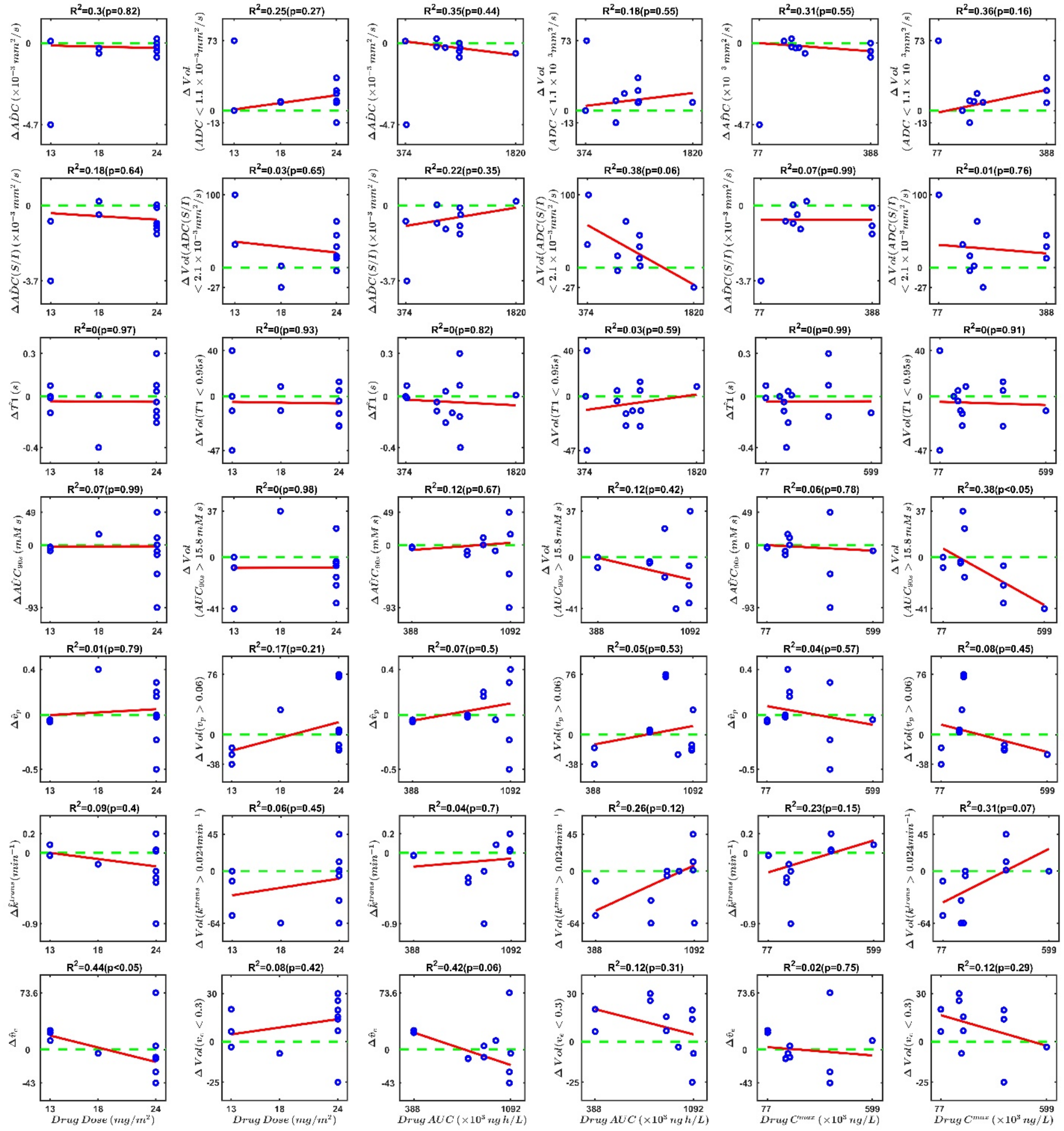

Figure A2: Linear regressions of mean changes and volume subpopulation changes at the core tumor per parameter vs. drug dose, drug AUC, and drug C^{max}.

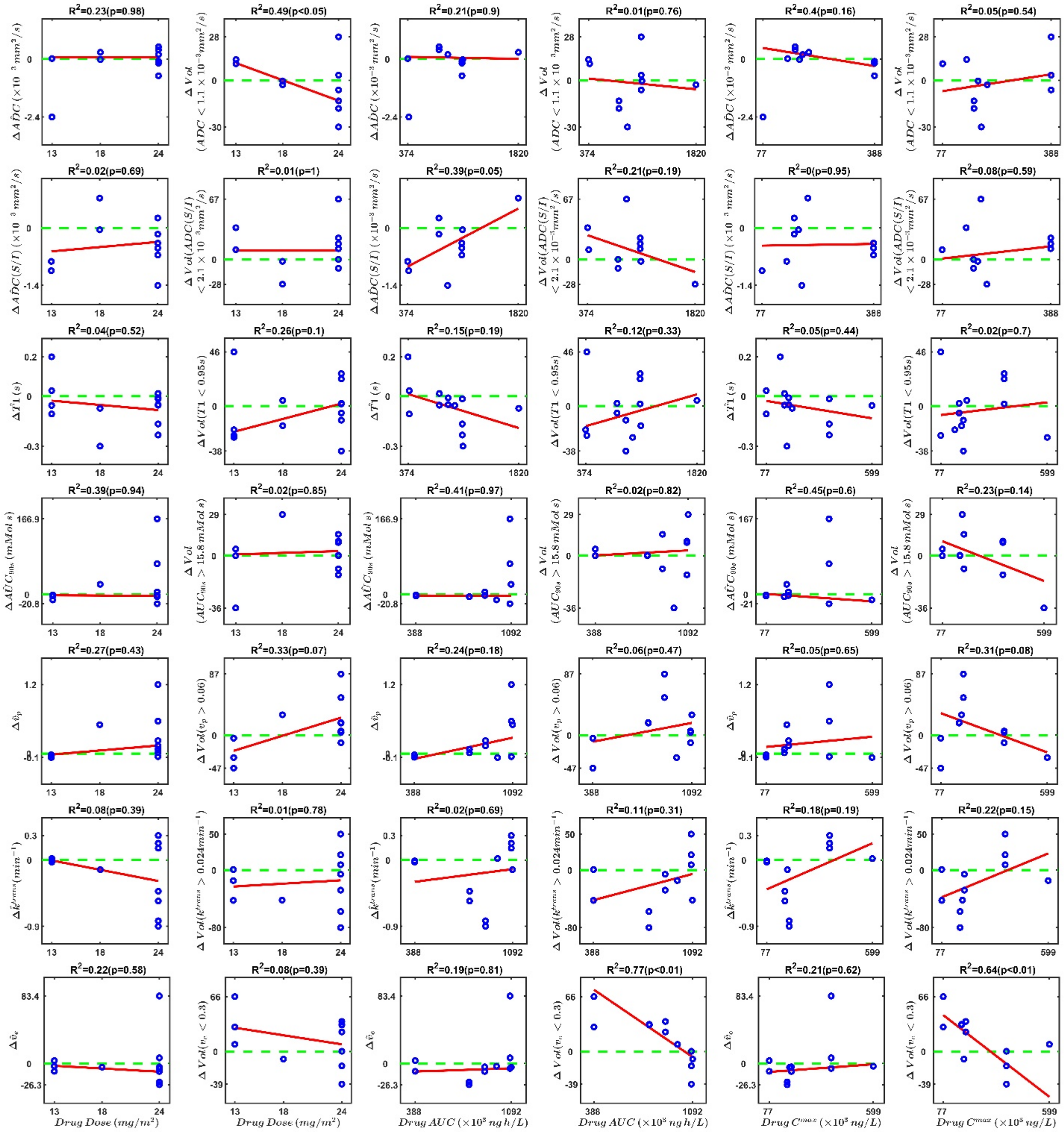

Figure A3: Linear regressions of mean changes and volume subpopulation changes at the rim tumor per parameter vs. drug dose, drug AUC, and drug C^{max}.

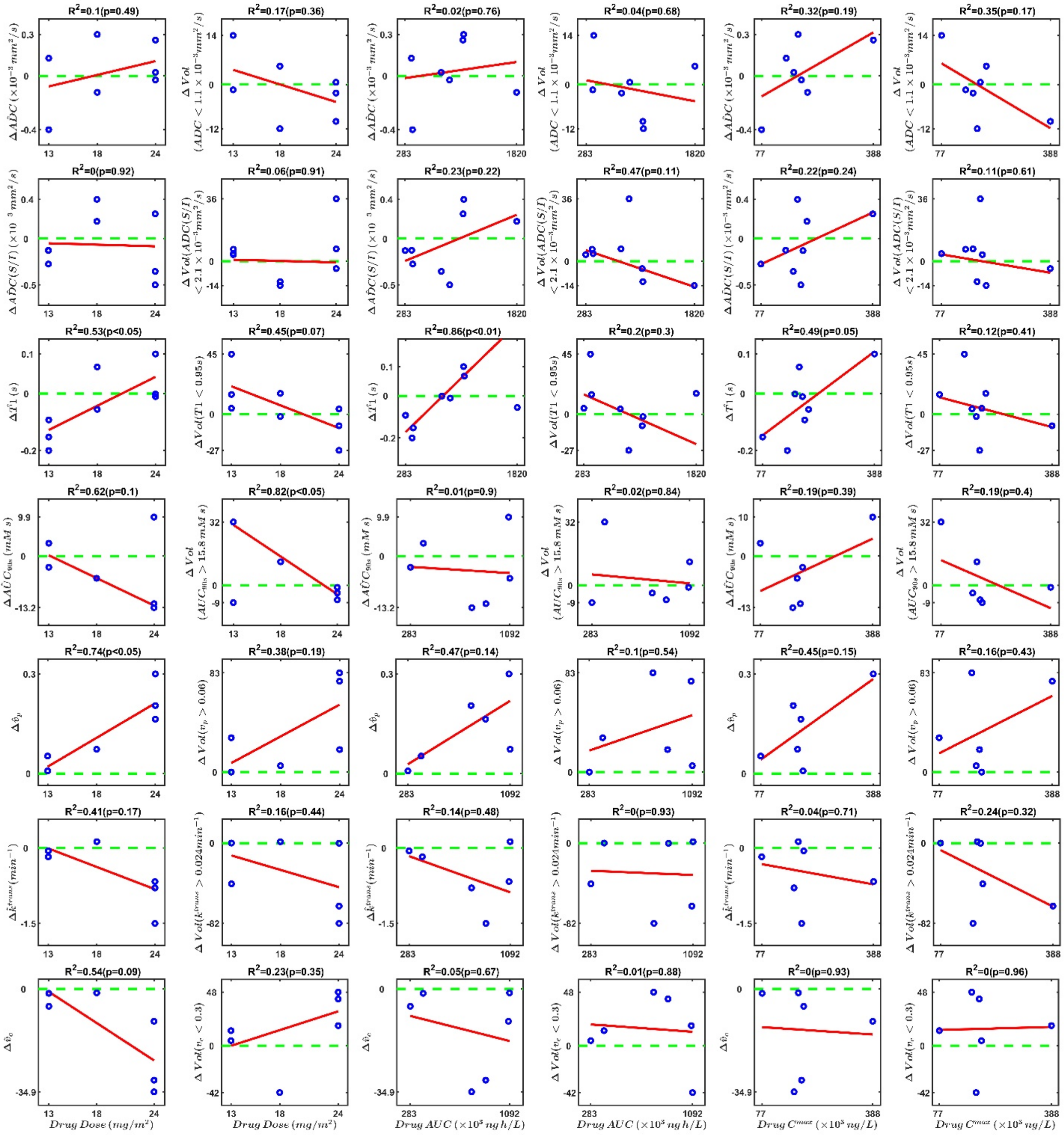

Figure A4: Linear regressions of mean changes and volume subpopulation changes at the liver per parameter vs. drug dose, drug AUC, and drug C^{max}.

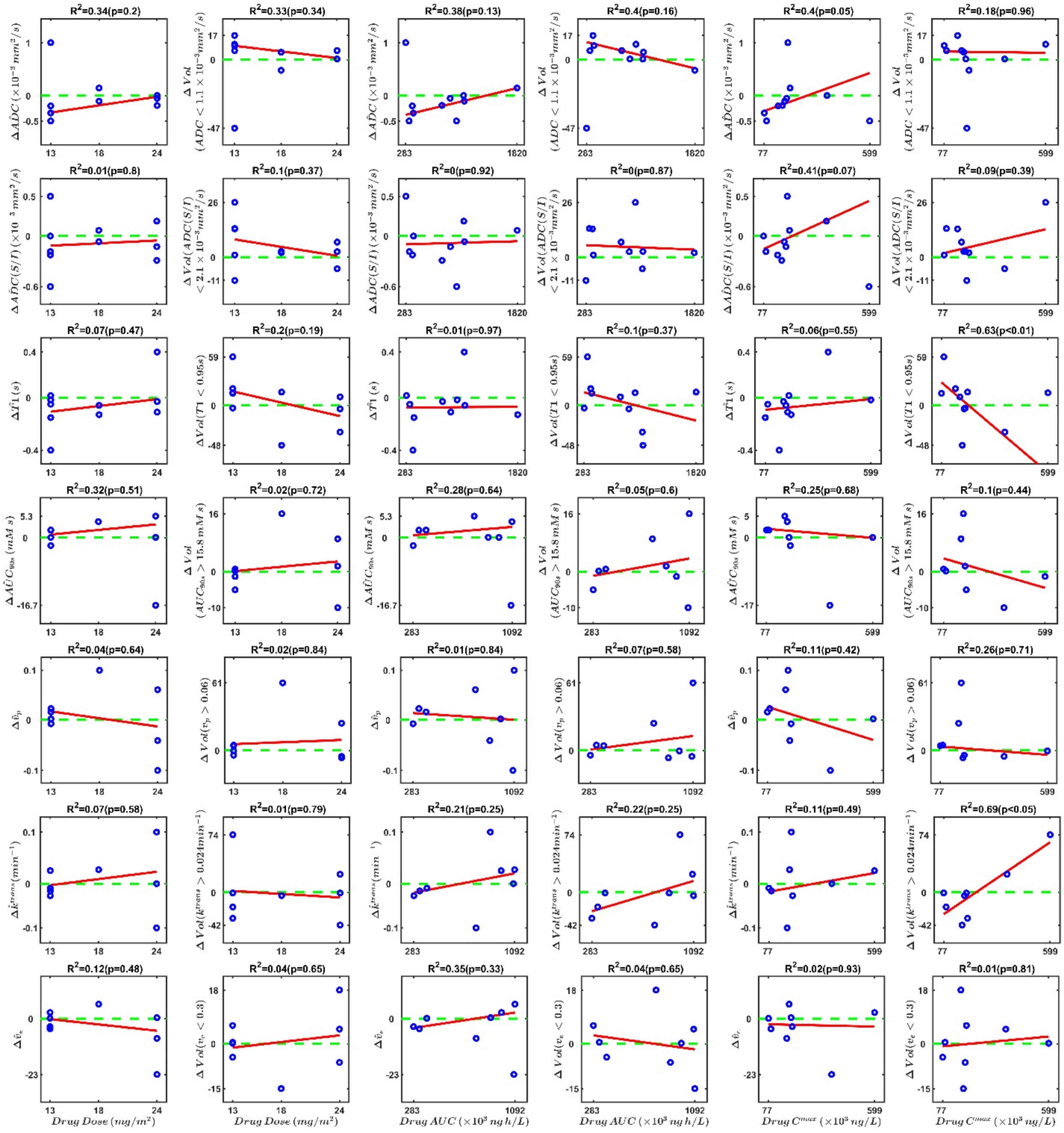

Figure A5: Linear regressions of mean changes and volume subpopulation changes at the muscle per parameter vs. drug dose, drug AUC, and drug C^{max}.

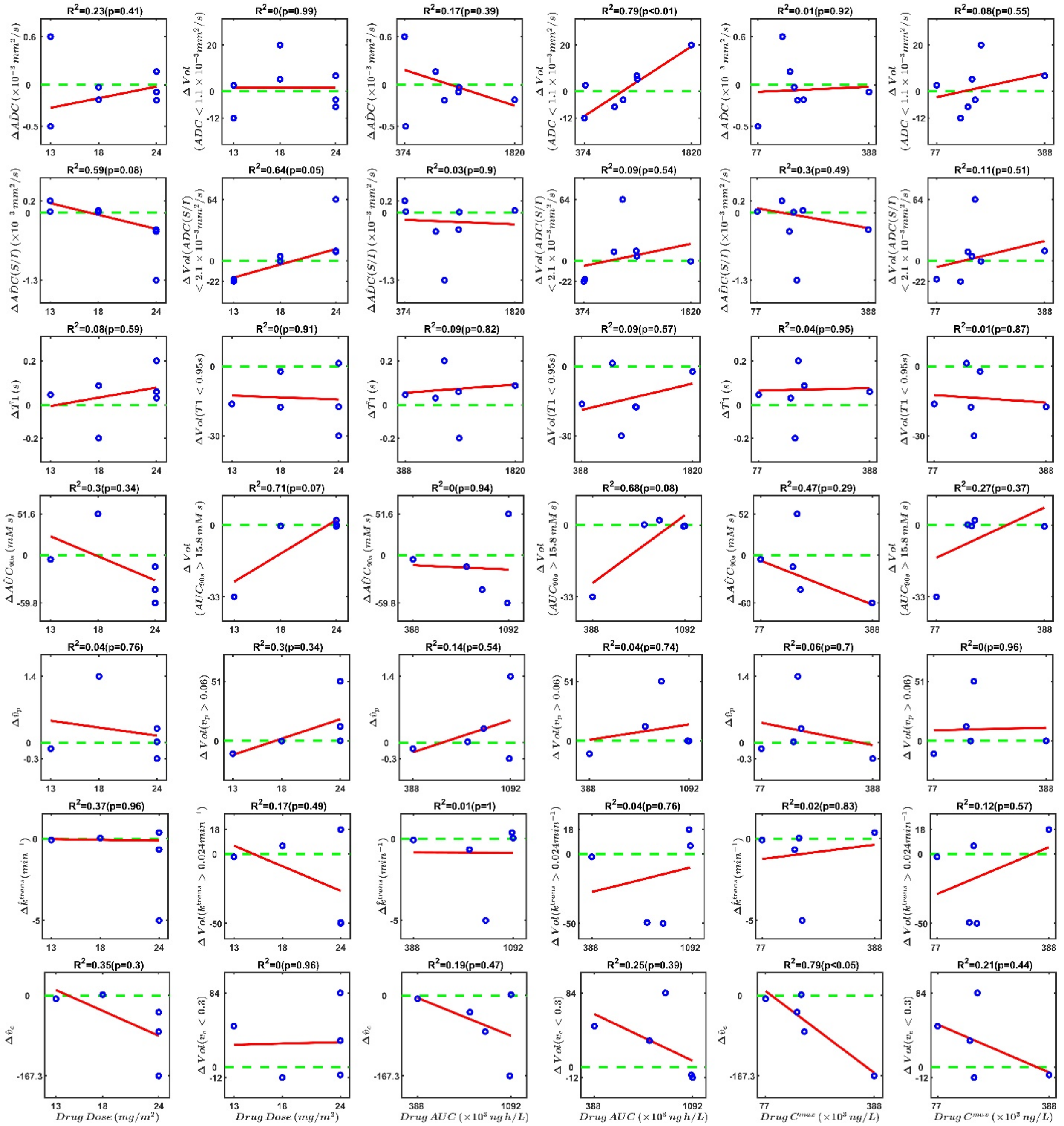

Figure A6: Linear regressions of mean changes and volume subpopulation changes at the spleen per parameter vs. drug dose, drug AUC, and drug C^{max}.

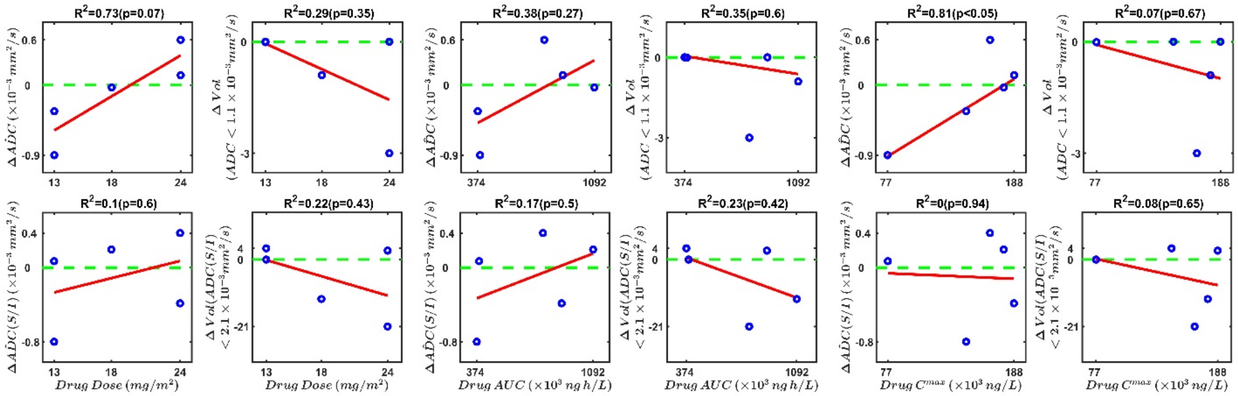

Figure A7: Linear regressions of mean changes and volume subpopulation changes at the renal cortex per parameter vs. drug dose, drug AUC, and drug C^{max}.
